# The Role of Phytochemicals and Plant-Based Diets in Gestational Diabetes: Evidence from Clinical Trials

**DOI:** 10.3390/ijerph20054188

**Published:** 2023-02-26

**Authors:** Kataryna Jaworsky, Pamela DeVillez, Arpita Basu

**Affiliations:** 1 Department of Kinesiology and Nutrition Sciences, School of Integrated Health Sciences, University of Nevada, Las Vegas, NV 89154, USA; 2 Kirk Kerkorian School of Medicine, University of Nevada, Las Vegas, NV 89154, USA

**Keywords:** gestational diabetes, phytochemicals, hyperglycemia, polyphenols

## Abstract

Gestational diabetes mellitus (GDM) is defined as glucose intolerance identified during pregnancy. The increased risk of pregnancy complications and the adverse health effects for the mother and baby associated with GDM require urgent and effective ways to control the condition. The primary goal of this semi-quantitative review was to examine the effects of phytochemicals and plant-based diets on GDM in clinical studies involving women undergoing pregnancy and to summarize the findings for application in clinical practice and disease management. The articles included in this review show that intervention strategies, including fruits, vegetables, whole grains, nuts and seeds, and tea, may be beneficial in the management of GDM and lower blood glucose and improve adverse pregnancy outcomes in these women. The randomized controlled trials reviewed collectively show improved glycemic control markers, blood lipid values, and body weight and composition when supplemented with phytochemical-rich supplements and foods compared to those in the control groups. The findings support the clinical observations of lower GDM risks in women consuming plant-derived diets rich in phytochemicals. Nutrition interventions involving plant-based foods and diets are thus a practical way to reduce hyperglycemia both in patients diagnosed with GDM and those at high risk for the development of GDM.

## 1. Introduction

Gestational diabetes mellitus (GDM) is defined as glucose intolerance identified during pregnancy [1]. The prevalence of GDM is difficult to estimate and varies among studies and countries. Women may have undiagnosed diabetes before pregnancy, which can play a role in skewed GDM rates [1]. Diabetes Research and Clinical Practice reports GDM prevalence from 1% to 14% annually in the U.S. [1]. According to Wang et al. (2021), GDM prevalence ranges from 6% to 8% of all pregnancies in the U.S. [2]; in Europe, its prevalence ranges from 8% to 52% [3]; China has a prevalence of approximately 8% [4]; Belgium, Italy, and the U.K. have a prevalence of 16%, 21%, and 24%, respectively [3]; Denmark has the highest prevalence at 52% [3]. As being overweight and obese during pregnancy are established risk factors for GDM, the rise in obesity may explain the concomitant rising prevalence of GDM [5]. Approximately 35% of women of reproductive age are obese in the U.S. [6]. In the U.S., the cost of maternal obesity is estimated at $106.8 million annually [6].

Plant-based diets and plant-derived dietary bioactive compounds, especially those derived from fruits, vegetables, whole grains, legumes, and various edible herbs and botanicals have been shown to reduce risks of diabetes and its complications [7]. Phytochemicals, or phytonutrients, are bioactive compounds found in foods such as vegetables, fruits, whole grains, nuts and seeds, and tea [8]. Dietary fruits, such as acai, goji berries, blueberries, and strawberries have high levels of antioxidants, fibers, vitamins, minerals, and phytochemicals [9]. Common phytochemicals include phytoestrogens, phenolic acids, flavonoids, carotenoids, polyphenols, indoles, isoflavones, stilbenoids, isothiocyanates, saponins, phenylpropanoids, and ginsenosides [8,9]. These phytochemicals have been associated with decreased risks of diabetes [7]. Research has identified several health benefits of bioactive phytochemicals, including antioxidant and anti-inflammatory activities, as well as normalizing glucose metabolism [8,9].

Pregnant women with obesity who develop GDM have approximately a 70% chance of developing type 2 diabetes later in life along with pregnancy complications [6]. There is an association between GDM and an increased risk of adverse pregnancy outcomes, such as macrosomia, premature birth, hypoglycemia at birth, and neonatal jaundice [10]. Additionally, infants are more often born with congenital abnormalities and are born large for gestational ages at birth to women with GDM [6]. Children born to mothers with obesity and GDM have an increased risk of being overweight and insulin resistant themselves, creating a cycle of metabolic disorders [6]. The risk of pregnancy complications and the adverse health effects for the mother and baby associated with GDM along with health care costs results in a need for urgent and effective intervention strategies for GDM.

Evidence is needed regarding intervention strategies to reduce GDM’s prevalence. Nutrition education is proven effective for improving glycemic control and other diabetes-related outcomes in type 2 diabetes [11]. Hashim et al. (2021) reported that educating on eating vegetables showed reduced glycosylated hemoglobin (HbA1c) levels in type 2 diabetes patients [11]. This leads to the importance of nutrition education in women with GDM. Research has shown promise that phytochemicals found in fruits and vegetables and plant-based diets lower blood glucose levels, and this is leading the way for future GDM intervention strategies [7]. In this semi-quantitative review, the effects of phytochemicals and plant-based diets on GDM in clinical studies involving pregnant women either diagnosed with GDM or at high risk for GDM are examined.

## 2. Methods

**Figure 1:**
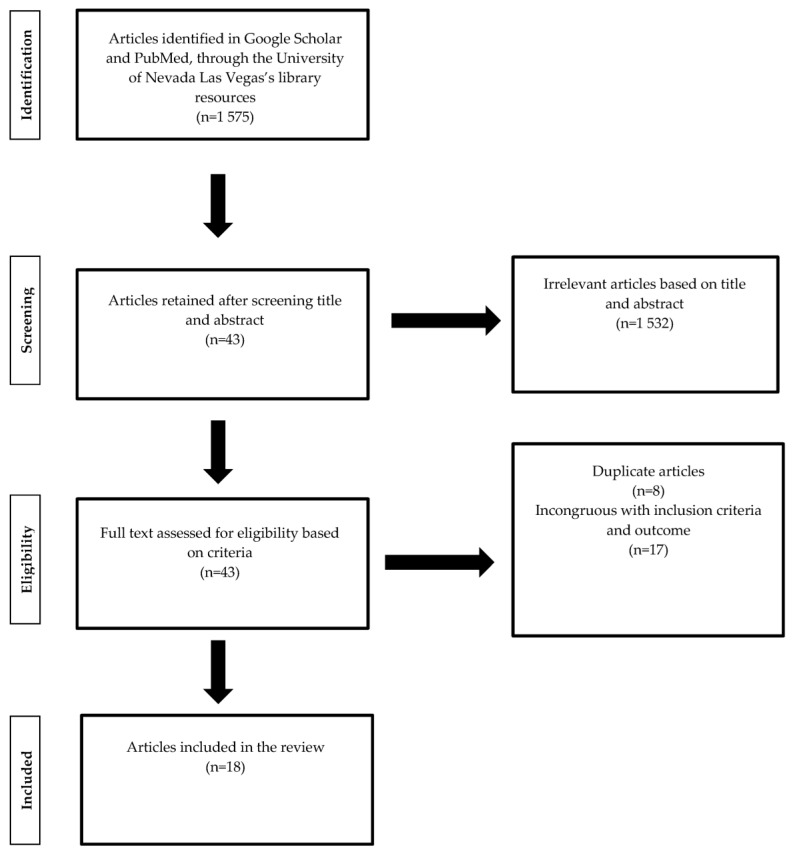
Literature review search process.

A literature search was conducted using databases, including PubMed and Google Scholar, through the University of Nevada Las Vegas’s library resources. Inclusion criteria included articles published between 2011 and 2021. Search keywords included “clinical trials” and “phytochemicals”, “plant-based diets”, “DASH diet”, “Med Diet”, “phytosterols”, “polyphenols”, “flavonoids”, “beta-carotene”, “isoflavones”, “phenolic acid”, “anthocyanin”, “vegetables”, “fruits”, “soy diets”, and “gestational diabetes mellitus”. Titles and abstracts were assessed to determine eligibility. After initially determining eligibility, articles’ full text was assessed to double-check inclusion eligibility. Inclusion criteria included human clinical trials and randomized clinical trials (RCTs). Inclusion of RCTs involved patients either with diagnosed GDM or at high risk for GDM and dietary intervention with randomized assignment to the experimental and control/placebo groups. Exclusion criteria for RCTs included non-human models and in vivo studies. Ultimately, 18 articles were eligible and included in this review (Figure 1).

For our semi-quantitative analysis, we reported calculated mean percent changes, along with the highest and lowest percent changes, for each outcome measure using the data reported in the results section (Section 3) of articles included in this review.

## 3. Results

All 18 studies included in this semi-quantitative literature review were RCTs and thus each study had its own unique control group to which the interventional group was compared (Table 1 and Table 2). All control groups consisted of pregnant women either with GDM or at high risk of GDM and were given a certain diet to follow per the specific study’s protocol.

### 3.1. Plant-Based Food Groups, Diet and GDM

#### 3.1.1. Blood Glucose Markers

Nine of the clinical trials examining the effects of plant-based diets and food groups on risks and outcomes and GDM measured fasting glucose levels (Table 1) [12,13,14,15,16,17,18,20,21]. Intervention groups had lower fasting glucose values compared to the control groups. The intervention groups had an average decrease in fasting glucose values of 9.51% (highest: 19.99%, lowest: 0.48%) [12,17], while the control groups had an increase of 19.90% (highest: 68.70%, lowest: 1.52%) [15,18]. One study reported a 0% change in the control groups fasting glucose values [17]. Three of the clinical trials reported HbA1c levels [14,16,20]. The intervention groups had an average of 3.36% decrease in HbA1c levels (highest: 4.55%, lowest: 2.17%) compared to the control groups, which had an average of 6.94% increase in HbA1c levels (highest: 15.55%, lowest: 1.34%) [14,20].

#### 3.1.2. HOMA-IR

Four of the clinical trials that focused on food groups and GDM outcomes reported Homeostatic Model Assessment of Insulin Resistance (HOMA-IR) (Table 1) [14,16,18,21]. Intervention groups consuming plant-based diets and food groups had lower HOMA-IR values compared to the control groups. The intervention groups reported an average decrease of 26.92% (highest: 34.33%, lowest: 19.51%) compared to the control groups, which reported an average increase of 45.74% (highest: 62.79%, lowest: 4.55%) [14,16,18,21].

#### 3.1.3. Blood Lipids

Three clinical trials reported blood lipid levels (Table 1) [14,18,20]. High-density lipoprotein (HDL) values for intervention groups averaged a 3.44% increase from baseline levels (highest: 6.70%, lowest: 0.17%) [18,20]. One study showed no change in HDL for the intervention group [14]. The control groups averaged a 6.54% increase (highest: 8.93%, lowest: 4.14%) [14,18]. The studies showed on average that low-density lipoprotein (LDL) values increased by 9.11% for intervention groups (highest: 16.22%, lowest: 2.00%) and increased by 10.55% for the control groups (highest: 23.16%, lowest: 0.90%) [14,18].

### 3.2. Plant-Based Supplements and GDM

#### 3.2.1. Blood Glucose Markers

Seven studies measured the effect of various plant-based supplements on fasting plasma glucose (FPG) levels in women diagnosed with GDM (Table 2) [22,23,24,25,27,28,29]. The control groups in 4 of these studies had an average 2.82% decrease in their FPG levels at the end of the study (highest: 6.98%, lowest: 0.84%) [22,23,25,27], and one study’s control group had a 1.14% increase in their FPG level at the end of the study [24]. The intervention groups of all five studies showed a significant decrease in the FPG levels with an average 12.95% decrease (highest: 25.16%, lowest: 4.06%) [22,23,24,25,27]. Two studies reported no significant changes in the FPG between the control and intervention groups in their respective studies [28,29].

One study did not report the FBS values at the end of the study duration, so no comparison of FPG is able to be made for this study [26]. This study did, however, report a significant difference in the insulin dose needed between the two groups, with the control group experiencing a 2.64% decrease in insulin dose and the intervention group experiencing a 31.91% decrease in insulin dose needed by the end of the trial [26].

Only one study reported changes in HbA1c levels after their respective intervention [25]. The control group in this study experienced a 1.47% increase in their HbA1c level at the end of the study, while the intervention group experienced an 11.59% decrease in their HbA1c level [25].

#### 3.2.2. HOMA-IR

Five studies reported the effects of their respective plant-based supplements on the HOMA-IR levels of their participants (Table 2) [22,24,25,27,28]. Three of these studies reported an increase in HOMA-IR levels in the control groups, with an average increase of 12.56% (highest: 26.67%, lowest: 2.92%) [22,25,27]. One study had a decrease of 5% in the HOMA-IR of the control group [24], and one study had a 0% change in the HOMA-IR of the control group when comparing the HOMA-IR at the end of the trial to the baseline levels [28]. All 5 studies reporting HOMA-IR levels showed a significant decrease in the HOMA-IR levels in the intervention groups with a 42.75% reduction in HOMA-IR at the end of the trial as compared to baseline levels (highest: 56.41%, lowest: 17.39%) [22,24,25,27,28].

One study did not report a baseline value of HOMA-IR in order to calculate a percent change but did find a significant difference between the HOMA-IR levels between the control and intervention groups at the end of the study, with the intervention group having a 15.12% lower HOMA-IR as compared to the control group [29].

#### 3.2.3. Blood Lipids

Three studies investigated the effects of their plant-based supplement interventions on the blood lipid levels of the participants (Table 2) [25,27,28]. HDL levels in the intervention group averaged a 16.72% increase from baseline (highest: 25.48%, lowest: 7.95%), while HDL levels in the control group averaged a 9.85% decrease (highest: 10.67%, lowest: 9.03%) [25,27]. Intervention groups experienced an average 17.91% decrease in their LDL levels (highest: 20.15%, lowest: 15.67%), and the control groups showed an average 10.46% increase in their LDL levels by the trial’s end (highest: 10.51%, lowest: 10.41%) [25,27]. One study reported no significant difference in both LDL and HDL levels between the control and intervention at the end of the study [28]. All three studies demonstrated the significant effects of their intervention on total cholesterol levels, with an average decrease of 18.47% in total cholesterol (highest: 20.64%, lowest: 14.69%) [25,27,28]. Two of the studies’ control groups experienced an average 11.91% increase in total cholesterol by the end of the trial (highest: 11.91%, lowest: 8.86%) [25,27], and one study’s control group showed a 2.08% decrease in total cholesterol by the end of the trial [28]. Finally, two studies reported significant changes in triglyceride levels of the GDM participants [27,28]. The control groups for each study showed varying trends, with one study’s control group experiencing a 19.57% increase in triglycerides by the end of the trial [27], and the other study’s control group showing a 1.79% decrease in their triglyceride levels [28]. Both studies showed a significant decrease in the triglyceride levels for their intervention groups at the end of the study, with an average 19.62% decrease in triglyceride levels compared to baseline (highest: 28.94%, lowest: 10.31%) [27,28].

## 4. Discussion

GDM necessitates a multifocal approach to management, often including a combination of pharmacologic management along with lifestyle modifications in the form of diet and exercise changes. Studies have begun to examine the effect of plant-based diets and plant-based supplements on the management of GDM. However, there is a lack of a cohesive review and summarization of the results of these studies; hence, the goal of this review was to examine the effects of plant-based diets and phytochemicals on glycemic control in women both with GDM and at high risk of GDM and to summarize the findings for clinical practice and disease management. Our review demonstrates that dietary intervention strategies, including plant-based diets and diets supplemented with additional phytochemicals, may be beneficial in the management of GDM via improved glycemic control and improved pregnancy outcomes in these women.

### 4.1. Plant-Based Diets and Food Groups

The ten RCTs examined for the effects of plant-based diets on GDM as compared to control diets demonstrated significant improvements in glycemic control markers in women with or at high risk for GDM [12,13,14,15,16,17,18,19,20,21]. The dietary interventions that involved supplementing with whole foods, fiber, fruits, vegetables, whole grains, nuts, and seeds, all of which are rich in beneficial phytochemicals and nutrients, are effective in reducing blood sugar levels in individuals [12,13,14,15,16,17,18,19,20,21]. The intervention groups in the nine studies reporting significant effects on fasting glucose levels had an average 9.5% decrease while the control groups experienced an average 19.9% increase in fasting glucose, suggesting that dietary modifications to include more plant-based options can be beneficial in improving glycemic control [12,13,14,15,16,17,18,20,21]. Both Zamani et al. (2019) and Chen et al. (2021) demonstrated that greater adherence to a plant-based diet decreased the risk of GDM development during pregnancy [30,31]. Moreover, Zamani et al. (2019) reported that plant-based diets higher in unhealthy alternatives, characterized by increased intake of fruit juices, sugar-sweetened beverages, refined grains, potatoes, and desserts, had a higher risk of GDM [30]. The results of this study correlate with the findings of this review in that increased dietary control via healthy plant-based methods promotes better glycemic control in pregnant women.

Furthermore, there was an average 3.36% decrease in HbA1c levels in the intervention group prior to dietary intervention compared to a 6.94% increase in the control group [14,16,20]. HbA1c is a measurement of the average glycosylated hemoglobin over the previous approximately 90 days. This is important regarding GDM as most patients are diagnosed around the 24–28 gestational week and will often deliver around 37–40 weeks; thus, comparing HbA1c over this period gives a snapshot in time that likely includes only the period in which dietary intervention was initiated, highlighting that much of the effect on HbA1c is, in part, directly due to the dietary changes. The positive effects on HbA1c that are demonstrated in this review are echoed by the RCT conducted by Lee et al. (2016) who focused on comparing a plant-based vegan diet to a conventional glycemic control diet for the management of HbA1c in type 2 diabetics [32]. They found that both the vegan diet and the glycemic control diet decreased HbA1c in these diabetic patients, but the vegan diet showed a significantly larger reduction in HbA1c as compared to the glycemic control diet, suggesting that a plant-based diet may confer an additional benefit in the glycemic management of patients with diabetes [32]. Many foods that encompass a healthy plant-based diet are high in fiber, which is one dietary factor that has been studied extensively regarding glycemic control. Reynolds et al. (2020) demonstrated in their meta-analysis that increased fiber intake resulted in reduced HbA1c and better overall glycemic control in individuals with both type 1 and type 2 diabetes [33]. While GDM patients were not included in this review since there is a paucity of RCTs that directly analyzed fiber supplementation in GDM glycemic control, it can be inferred that the mechanism of fiber supplementation and improved glycemic control can apply to gestational diabetes as well given a similar cause of insulin resistance.

HOMA-IR, a calculation tool that assesses pancreatic B-cell function and insulin resistance via a relationship between fasting plasma glucose and fasting plasma insulin, is another important measurement in glycemic control. Intervention trials that incorporated plant-based foods and dietary models into their daily diet reported an average 26.9% decrease in HOMA-IR scores as compared to a 45.7% increase in HOMA-IR scores in the control group [14,16,18,21]. Lower HOMA-IR scores are suggestive of decreased insulin resistance and thus increased sensitivity to insulin, whereas higher HOMA-IR scores suggest increased insulin resistance. Therefore, the addition of plant-based dietary changes demonstrated more insulin sensitivity in the intervention group, indicating that plant-based diets influence insulin sensitivity which then goes on to affect blood glucose levels as well. The effects of plant-based diets on insulin have been well-documented- Kahleova, et al. (2020) demonstrated in their randomized control trial that implementation of a low-fat vegan diet for 16 weeks resulted in a decreased HOMA-IR score for the intervention patients, suggesting improved insulin sensitivity in these type 2 diabetic patients [34]. This study highlights that even a plant-based dietary change for 16 weeks, a relatively short amount of time that is similar to the amount of time that GDM patients would have to make the dietary change as they are diagnosed around 24 weeks of gestation and deliver around 40 weeks gestation, is beneficial to the glycemic control of the patient [34]. In addition, a review article by Adeva-Andany et al. (2019) demonstrated that diets high in plant-based foods enhance insulin sensitivity, mirroring the findings of this review [35]. They also demonstrated that pre-pregnancy diets high in meat and low in plant-based foods are a hallmark for insulin resistance during pregnancy, suggesting that even prepartum dietary habits can influence the development of GDM during future pregnancy [35].

Finally, multiple influences of the plant-based dietary changes on cholesterol levels were reported in the RCTs. HDL values, more colloquially known as the “good cholesterol” due to their beneficial effects in removing cholesterol from the peripheral tissues and returning it to the liver for processing, increased by 3.44% from baseline on average in the intervention groups [14,18,20]. Interestingly, the control groups in the studies reporting HDL levels experienced a greater increase in HDL levels at an average of 6.54% [14,18,20]. LDL levels on average increased by 9.11% for the intervention groups and by 10.55% for the control groups [14,18]. The BROAD study by Wright et al. (2017) demonstrated that a whole-food plant-based diet resulted in significant improvements in cholesterol levels in patients diagnosed with diabetes, obesity, and ischemic heart disease, all of which are diseases modified by cholesterol levels [36]. Similar trends with decreasing cholesterol and increased glycemic controls have been reported, but these studies on pregnant women with gestational diabetes, other than the ones reported in this review, are lacking. Our review offers support for the effect of plant-based diets on cholesterol management specifically in GDM.

### 4.2. Plant-Based Supplements

Eight RCTs examined the influence of specific plant-based supplements, including ginger, garlic, EGCG, phytosterols, L-ascorbic acid, chili powder, and soybean oligosaccharides, on GDM markers and subsequently demonstrated that these supplements had a significant effect on at least one, and often multiple, markers of GDM outcomes [22,23,24,25,26,27,28,29]. Seven of these studies reported significant effects of these supplements on fasting plasma glucose, with the intervention groups demonstrating a 12.9% decrease in fasting plasma glucose [22,23,24,25,27]. The control groups’ data were more widespread with 4 studies showing an average decrease of 2.8% and one study showing a 1.1% increase in fasting glucose levels at the end of the study periods [22,23,24,25,27]. The findings of this review mirror the results reported by Zhu et al. (2021) who elucidated the mechanism of phytochemicals, mainly through teas with high levels of active phytochemicals such as catechin, epicatechin, epicatechin gallate, and epigallocatechin gallate (EGCG), in alleviating insulin resistance via hypoglycemic activity, inhibiting glycosidase, and reducing oxidative stress in mice [37]. Given that one study in our review reported EGCG and its positive effects on glycemic control, we can postulate that a similar mechanism of these phytochemicals is at play with the plant-based phytochemical supplementation in GDM control. Similarly, An et al. (2020) reported in their review that oligosaccharide supplementation resulted in improved glycemic control via decreased fasting glucose, HbA1c, and HOMA-IR scores, showing similar results to our review [38]. Finally, Tabatabaei-Malazy et al. (2014) demonstrated in their meta-analysis that supplementation of L-ascorbic acid, a form of vitamin C and a powerful antioxidant, alone resulted in a significant decrease in fasting blood sugar in type 2 diabetes [39]. These findings suggest that ascorbic acid supplementation through vitamin C, something readily available in most pharmacies and grocery stores, can assist in decreasing fasting blood sugar and can assist in improving glycemic control. Vitamin C is often already included in the prenatal vitamins that pregnant patients are encouraged to take throughout their pregnancy, thus encouraging the use of prenatal vitamins, and potentially increasing supplementation of ascorbic acid specifically in GDM patients, should be considered clinically.

Only one study reported the effects of HbA1c with regard to plant-based supplement use, specifically phytosterols. This study showed an 11.59% decrease in the HbA1c in the intervention group and a 1.14% increase in the control group’s HbA1c [26]. A meta-analysis conducted by Salehi-Sahlabadi et al. (2020) reported that phytosterol supplementation of 1–2 g daily, a similar dose to the study reported in this review, resulted in significantly lower HbA1c levels and better overall glycemic control measured via fasting blood glucose [40]. However, as Salehi-Sahlabadi mentions in their review, phytosterols are present in daily foods, such as fruits, and yogurts, and are often added to margarine, but achieving 1–2 g daily in habitual diets is difficult and would require active supplementation, something that could be considered clinically for the management of GDM [40].

HOMA-IR scores were reported by five of the studies examining specific plant-based supplements and there was an average 42.75% reduction in HOMA-IR scores compared to baseline by the end of the trial, suggesting that the specific plant-based supplements analyzed in these studies (ginger, EGCG, phytosterols, chili powder) may result in increased insulin sensitivity and thus better glycemic control [22,24,25,27,28]. Maharlouei et al. (2019) conducted a meta-analysis looking at the effect of ginger, one of the significant plant-based supplements in our review, on various markers of metabolic and glycemic control and found that increased ginger supplementation had a host of benefits, including a significantly decreased HOMA-IR score suggesting improved insulin sensitivity [41]. This can translate into the GDM patient population as ginger is a more readily available supplement that can easily be added to the diet for increased glycemic and lipid control. Chili powder is an additional plant-based supplement that is easily accessible to the general population that has been demonstrated to have positive effects on insulin sensitivity- Li et al. (2014) found that individuals who reported no intake of chili-containing foods had significantly higher HOMA-IR scores, and thus higher insulin resistance, as compared to individuals with high intake of chili-containing foods [42]. The mechanisms for many of these dietary supplements and their effect on glucose metabolism are still being elucidated, but recent studies have shown that capsaicin, the major active constituent in chili powder, has a direct effect on multiple metabolic tissue pathways. Specifically, Panchal et al. (2018) summarized that capsaicin activated many metabolic modulators including glucagon-like peptide 1 (GLP-1), which is a protein directly involved in glucose metabolism via stimulation of insulin release and thus increased metabolism of glucose [43]. Many of these plant-based supplements examined in the articles included in this review likely have similar mechanisms for improving insulin sensitivity and thus more research into these supplements and potential implementation in dietary control of insulin resistance is needed.

Finally, numerous effects on blood lipids by the various plant-based supplements were reported across the RCTs reviewed. HDL levels in the intervention group averaged a 16.72% increase from baseline while HDL levels in the control group averaged a 9.85% decrease [25,27]. Intervention groups experienced an average 17.91% decrease in their LDL and the control groups showed an average 10.46% increase in their LDL levels by the trial’s end [25,27]. Total cholesterol exhibited an average decrease of 18.47% in intervention groups [25,27,28]. These findings parallel the findings reported by Lee et al.’s (2003) RCT looking at phytosterol-enriched spreads in the lipid profile of type 2 diabetics [44]. They reported that increased phytosterols in diet were effective in reducing both total and LDL cholesterol and can be considered as a supplement for both increased glycemic and lipid control in patients with diabetic-related diseases, including GDM [44]. Finally, there was a documented significant decrease in the triglyceride levels for intervention groups at the end of the study, with an average 19.62% decrease in triglyceride levels compared to baseline [27,28]. Wang, et al. (2019) demonstrated that phytosterol supplementation in the diet of type 2 diabetics resulted in a significant decrease in triglycerides as compared to the control group, indicating that the addition of phytosterols into the diet of individuals with impaired glucose regulation, such as GDM patients, would be beneficial in improving lipid metabolism [45].

### 4.3. Strengths and Limitations

This review paper has several strengths. First, this semi-quantitative review offers a comprehensive examination of many published studies regarding plant-based diets and dietary supplementation and their role in GDM management. While this is an area that is becoming more widely studied, there was a lack of articles that summarized the major findings (hence the importance of this review). Secondly, all studies included in this review article were all RCTs, all of which had a control or placebo group. Thirdly, a wide variety of plant-based diets and supplementations were able to be examined in this review. For example, of the eight studies looking at plant-based supplements, seven unique supplements were studied, providing a wide variety of potential supplements that can be used clinically. Finally, this review offers summative suggestions for plant-based dietary modifications and supplementation for additional glycemic control for pregnant women with or at high risk for GDM that may be more cost effective or easier to implement compared to pharmacologic management.

However, we acknowledge the limitations present within our review as well. Since this review serves the purpose of providing an overview of various dietary interventions on multiple outcomes in hopes of providing potential recommendations for the clinical management of GDM, this review was structured to be a semi-quantitative review and not a systematic review. In addition, the literature search was only conducted on PubMed and Google Scholar, and while these sources represent a large proportion of published research, it is still only a subset of all the research regarding plant-based dietary modifications and markers of GDM. Furthermore, statistical analyses to determine the significance of the relationships between dietary interventions and the outcomes measured (changes in blood glucose, weight changes during pregnancy, etc.) were not conducted in this study. While the lack of statistical significance is important to note, the clinical significance of the findings in this review is important as multiple types of dietary intervention were able to be analyzed, such as glycemic control and plant-based diets for example, both independently, with the results broken down by study in the reported tables, and as a whole, with the average percent changes calculated. Finally, it is important to note that 16 out of the 18 studies included in this semi-quantitative review were conducted in non-Western countries, with the majority being conducted in either Iran or China. These countries have different dietary and nutritional habits as compared to the United States or other European countries. Thus, it is possible that these different baseline dietary customs may account for some of the results of GDM management via dietary interventions and should not be overlooked. Despite these limitations, we still uphold that the results of this review provide important summative information on the effect of plant-based diets and dietary supplementation on markers of GDM and can be used both in the clinical setting and as a driver of future research.

### 4.4. Future Directions

While our article provides a necessary comprehensive review of the impact of plant-based diets and dietary supplementation on markers of GDM, there is still a wide breadth of research that can be conducted in this field. This review has shown that these types of dietary modifications make a true difference in GDM markers such as serum glucose and weight gain that provide beneficial effects for both the mother and fetus. Continued RCTs with larger, more diverse populations of women diagnosed with or at risk of GDM are important to determine the statistical, and ultimately clinical, utility of the dietary interventions implemented. In addition, fetal outcomes such as neonatal hypoglycemia, shoulder dystocia, and macrosomia could be followed in these studies, as there is a paucity of studies that report effects on both the mother and the newborn. Finally, well-conducted systematic reviews and meta-analyses looking at the plant-based dietary intervention effects on GDM markers will provide precise data to both clinicians and expectant mothers on how these dietary interventions can affect their pregnancy and outcomes.

## 5. Conclusions

The findings of this semi-quantitative review agree with previous research results that have examined the use of phytochemicals in animal models and in clinical trials of gestational diabetes. The current research data in women with or at high risk for GDM has shown that plant-based diets and phytochemicals may reduce blood glucose and improve antioxidant activities to reduce oxidative stress that is often associated with GDM. Nutrition interventions are a practical way to educate mothers on lifestyle changes to reduce blood glucose and the complications associated with GDM. The high prevalence of GDM creates a need for more research and effective nutritional intervention strategies that focus on educating women to consume phytochemical-rich foods to reduce pregnancy complications of GDM.

## Figures and Tables

**Table 1:** Clinical trials on plant-based diets and food groups and their effects on gestational diabetes mellitus risks and outcomes.

Authors, Year (Country)	Trial Design and Duration	Participants	Dietary Intervention	Control Group Diet	Blood Glucose	Blood Lipid Levels	Body Composition/ Body Weight
Barati, et al., 2021 (Iran) [12]	RCT; 4 weeks	Women with GDM (n = 104)	Standard GDM diet with 30 g oat bran with lunch and dinner	Standard GDM diet without oat bran	↓ FBS	NR	Not statistically significant from control group
Basu, et al., 2021 (US) [13]	Randomized parallel arm study; 18 weeks	Women at high risk of GDM (n = 34)	Supplemented with 2 cups whole blueberries and 12 g soluble fiber, dietary intakes recorded on 24 h food recall	Standard prenatal care based on USDA Dietary Guidelines for Americans for pregnant women	↓ Postprandial blood glucose	NR	Body weight gain ↓ in Intervention group than control group
Basu, et al., 2021 (US) [14]	RCT; 18 weeks	Women at high risk of GDM and BMI ≥ 30 (n = 34)	Biweekly supply of 280 g frozen blueberries for snack and 12 g soluble fiber daily	Standard prenatal care based on USDA Dietary Guidelines for Americans for pregnant women	↓ 1 h postprandial blood glucose ↑ Serum glucose ↑ Serum Insulin ↑ HOMA-IR ↓ Serum HbA1c	↑ Total Cholesterol ↑ LDL Cholesterol ↑ Serum Triglycerides	Body weight gain ↓ in Intervention group than control group
Feng, et al., 2019 (China) [15]	Randomized, controlled, crossover study; same-day testing	Women with GIGT or GDM (n = 55)	Provided 42 g Pistachios to consume within 15 min	Provided 100 g Whole Wheat Bread to consume within 15 min	↓ Blood glucose ↓ Blood Insulin	NR	NR
Assaf-Balut, et al., 2017 (Spain) [16]	Unicentric, clinic-based, prospective, randomized controlled trial with two parallel groups; 18 weeks	Women with FBG < 92 mg/dL (n = 874)	Same as control diet plus lifestyle provided with EVOO and pistachios, consumption of at least 40 mg EVOO and handful (25–30 g) pistachios	Basic MedDiet, ≥2 servings/day vegetables, ≥3 servings/day fruit (avoid fruit juice), 3 servings/day skimmed dairy products, whole grain cereals, 2–3 servings legumes/week, moderate–high fish consumption, low red and processed meat consumption, avoid refined grains, baked goods, soft drinks, fast foods, pre-cooked meals	↓ FBG ↓ 2 h post glucose load ↓ HbA1c ↓ HOMA-IR	NR	↓ GWG compared to control group
Sahariah, et al., 2015 (India) [17]	RCT; 32 weeks	Pregnant women at risk for GDM (n = 1008)	Snack containing leafy green vegetables in fresh (~30 g) or dried (~7.5 g) form, 12–16 g full-fat milk powder, and 4–60 g dried fruit, started pre-conception	Maintained regular diet	Fasting glucose, 120 min glucose, and fasting insulin are not statistically significant between groups	NR	Not statistically significant between groups
Jamilian, et al., 2015 (Iran) [18]	Randomized, parallel clinical trial; 6 weeks	Women with GDM (n = 68)	0.8 g/kg protein (35% animal, 35% soy, 30% other plants)	0.8 g/kg protein (70% animal, 30% plant)	↓ FPG ↓ HOMA-IR	↓ Total Cholesterol ↓ Triglycerides ↓LDL ↓ T/HDL-C ratio	Not statistically different from control
Asemi, et al., 2014 (Iran) [19]	Two-arm parallel RCT; 4 weeks	Women with GDM (n = 52)	Calorie and protein composition are similar to control. Rich in fruits, vegetables, whole grains, and low-fat dairy and low in saturated fats, cholesterol, refined grains and sweets	45–55% CHO, 15–20% protein, 25–30% total fat	↓ Insulin Therapy	NR	Not statistically different from control
Asemi, et al. 2013 (Iran) [20]	Two-arm parallel RCT; 4 weeks	Women with GDM (n = 34)	Similar to control and rich in fruits, vegetables, whole grains, low-fat dairy and low in saturated fats, cholesterol, refined grains and sweets. Sodium was 2400 mg/d	45–55% CHO, 15–20% protein, 25–30% total fat, 7 d menu cycle based on usual practice in GDM	↓ 1 h GTT ↓ 2 h GTT ↓ 3 h GTT ↓ HbA1c	↓ Total Cholesterol ↓ TAG ↑ HDL ↓ LDL ↓ T/HDL-C ratio	Not statistically significant from control
Asemi, et al., 2013 (Iran) [21]	Randomized, two-arm, parallel clinical trial; 4 weeks	Women with GDM (n = 32)	DASH diet is rich in fruits, vegetables, whole grains, low-fat dairy products and low in saturated fats, cholesterol, refined grains, and sweets. Sodium reduced to ˂2000 mg/d, 7 d menu cycle	Based on recommendations for acceptable dietary intake for GDM: 40–50% CHO, 10–20% Protein, 25–30% total fats	↓ FPG ↓ Serum Insulin ↓ HOMA-IR	↓ Total Cholesterol	Not statistically significant from placebo

Up (↑) and down (↓) arrows indicate an increase and decrease, respectively, in the outcome being measured between the intervention and control group. Abbreviations: BMI: body mass index; CHO: Carbohydrates; d: days; DASH: Dietary Approaches to Stop Hypertension; EVOO: Extra Virgin Olive Oil; FBS: Fasting Blood Sugar; FPG: Fasting Plasma Glucose; g: grams; GDM: Gestational Diabetes Mellitus; GIGT: Gestational impaired glucose tolerance; GTT: Glucose Tolerance Test; GWG: Gestational Weight Gain; h: hours; HbA1c: Hemoglobin A1c; HOMA-IR: Homeostasis Model of Assessment-Insulin Resistance; kg: kilograms; mg/dL: milligrams/deciliter; min: minutes; n: sample size; NR: Not reported; RCT: Randomized controlled trial; TAG: Triacylglycerides; T/HDL-C ratio: Total-cholesterol to HDL-C ratio; US: United States.

**Table 2:** Clinical trials on dietary plant-based supplements and their effects on gestational diabetes mellitus risks and outcomes.

Authors, Year (Country)	Trial Design and Duration	Participants	Dietary Intervention	Control Group Diet	Blood Glucose	Blood Lipid Levels	Body Composition/ Body Weight
Hajimoosayi, et al., 2020 (Iran) [22]	Double-blind, placebo-controlled RCT, 6 weeks	Women with GDM (n = 70), 24–28 weeks gestation	1500 mg ginger, split into 3 tablets	Placebo pill, split into 3 tablets	↓ FBS ↓ Serum insulin ↓ HOMA-IR	NR	Not significantly different from control group
Faroughi, et al., 2018 (Iran) [23]	Triple-blind, placebo-controlled RCT, 8 weeks	Women with prediabetes and borderline GDM (n = 44), 24–28 weeks gestation	1 pill with 400 mg dry garlic powder (1200–1800 mg allicin and 2 g fresh garlic)	1 placebo pill, made of starch	↓ FBS ↓ GDM incidence after intervention	NR	Not significantly different from control group
Zhang, et al., 2017 (China) [24]	Double-blind, placebo-controlled RCT, maintained until delivery	Women with GDM (n = 326), diagnosed at onset of 3rd trimester (29 wks)	1 capsule containing 500 mg EGCG	1 placebo capsule containing 500 mg starch powder	↓ FPG ↓ Serum insulin ↓ HOMA-IR ↓ HOMA-B ↑ QUICKI	NR	Not significantly different from control group
Gao, et al., 2017 (China) [25]	Double-blind, placebo-controlled RCT, maintained until delivery	Women with GDM (n = 276), diagnosed GDM at onset of 3rd trimester (29 wks)	Phytosterol-enriched margarine spread, 10 g serving of spread with 2 g of phytosterols, 2x daily	Regular margarine spread, 10 g serving, 2x daily	↓ FPG ↓ HbA1c ↓ Serum insulin ↓ HOMA-IR ↓ HOMA-B ↑ QUICKI	↓ Total Cholesterol ↓ LDL ↑ HDL ↓ TC/HDL ratio	Not significantly different from control group
Maged, et al., 2016 (Egypt) [26]	Placebo-controlled RCT, maintained until delivery	Women with GDM (n = 200)	1 g L-ascorbic acid effervescent tablet	1 placebo effervescent tablet	↓ Insulin dosage	NR	NR
Li, et al., 2016 (China) [27]	Double-blind, placebo-controlled RCT, 16 weeks	Women with GDM (n = 222), diagnosed GDM at onset of 2nd trimester (13 wks)	Phytosterol-enriched margarine spread, 10 g serving of spread with 2 g of phytosterols, 2x daily	Regular margarine spread, 10 g serving, 2x daily	↓ FPG ↓ Serum insulin ↓ HOMA-IR ↓ HOMA-B ↑ QUICKI	↓ Triacylglycerol ↓ Total Cholesterol ↓ LDL ↑ HDL ↓ TC/HDL ratio	Not significantly different from control group
Yuan, et al., 2016 (China) [28]	Double-blind, placebo-controlled RCT, 4 weeks	Women with GDM (n = 44), between 22–33 weeks gestation	0.625 g chili powder with 2.5 mg of capsaicin, 2x daily	0.625 g chili powder with 0 mg of capsaicin, 2x daily	↓ 2 h PG ↓ 2 h insulin ↓ 2 h HOMA-IR ↑ CGRP	↓ Total cholesterol ↓ Triglycerides ↑ Serum apolipoprotein B	Not significantly different from control group
Fei, et al., 2014 (China) [29]	Placebo-controlled RCT, 8 weeks	Women with GDM (n = 97), currently on insulin treatment	Soybean oligosaccharides (combination of raffinose, stachyose, and sucrose) 10 g in 200–300 mL water orally, along with 3x injection of NovoRapid Insulin (short-term) before meals with a 1x injection of Novolin N insulin (intermediate-term) before sleep	3x injection of NovoRapid Insulin (short-term) before meals with a 1x injection of Novolin N insulin (intermediate-term) before sleep	↓ Fasting insulin ↓ HOMA-IR ↑ Adiponectin ↓ Total insulin dosage	NR	NR

Up (↑) and down (↓) arrows indicate an increase and decrease, respectively, in the outcome being measured between the intervention and control group. Abbreviations: CGRP: calcitonin gene-related peptide; EGCG: epigallocatechin 3-gallate; FBS: fasting blood sugar; FPG: fasting plasma glucose; g: grams; GDM: gestational diabetes mellitus; HbA1c: glycated hemoglobin; HDL: high-density lipoprotein; HOMA-B: homeostasis model of assessment of β cell function; HOMA-IR: homeostasis model of assessment of insulin resistance; LDL: low-density lipoprotein; mg: milligrams; n: sample size; NR: not reported; PG: plasma glucose; QUICKI: quantitative insulin check index; RCT: randomized controlled trial; TC: total cholesterol; wks: weeks, #x: number of times intervention is taken daily.

## Data Availability

Not applicable.
